# Roles of the Functional Interaction between Brain Cholinergic and Dopaminergic Systems in the Pathogenesis and Treatment of Schizophrenia and Parkinson’s Disease

**DOI:** 10.3390/ijms22094299

**Published:** 2021-04-21

**Authors:** Srijan Acharya, Kyeong-Man Kim

**Affiliations:** 1 Pharmacology Laboratory, College of Pharmacy, Chonnam National University, Gwang-Ju 61186, Korea; pharma.srijan@gmail.com; 2 Department of Pathology, College of Medicine, University of South Alabama, Mobile, AL 36617, USA; 3 Cancer Biology Program, Mitchell Cancer Institute, University of South Alabama, Mobile, AL 36604, USA

**Keywords:** dopamine, nicotine, receptor, schizophrenia, Parkinson’s disease, L-DOPA

## Abstract

Most physiologic processes in the brain and related diseases involve more than one neurotransmitter system. Thus, elucidation of the interaction between different neurotransmitter systems could allow for better therapeutic approaches to the treatments of related diseases. Dopaminergic (DAergic) and cholinergic neurotransmitter system regulate various brain functions that include cognition, movement, emotion, etc. This review focuses on the interaction between the brain DAergic and cholinergic systems with respect to the pathogenesis and treatment of schizophrenia and Parkinson’s disease (PD). We first discussed the selection of motor plans at the level of basal ganglia, the major DAergic and cholinergic pathways in the brain, and the receptor subtypes involved in the interaction between the two signaling systems. Next, the roles of each signaling system were discussed in the context of the negative symptoms of schizophrenia, with a focus on the α7 nicotinic cholinergic receptor and the dopamine D_1_ receptor in the prefrontal cortex. In addition, the roles of the nicotinic and dopamine receptors were discussed in the context of regulation of striatal cholinergic interneurons, which play crucial roles in the degeneration of nigrostriatal DAergic neurons and the development of L-DOPA-induced dyskinesia in PD patients. Finally, we discussed the general mechanisms of nicotine-induced protection of DAergic neurons.

## 1. Introduction

Owing to the roles of the brain dopaminergic (DAergic) system in addiction, interactions between the nicotinic cholinergic and DAergic systems have been extensively studied with respect to nicotine addiction [1,2]. Application of nicotinic drugs or stimulation of cholinergic projections into the ventral tegmental area (VTA) induces dopamine (DA) release in the nucleus accumbens (NAc), which contributes to the addictive properties of cigarette smoking [2]. Among the multiple subtypes of nicotinic cholinergic receptors (nAChRs), α4β2* and α6β2* are the major subtypes involved in the attention-enhancing and pathophysiology of nicotine addiction [3,4].

Involvement of specific DA receptor subtypes has also been reported in drug addiction. For example, early nicotine exposure is accompanied by an increase in DA D_3_ receptor (D_3_R) expression [5], and D_3_R antagonists have been reported to be effective for the treatment of nicotine dependence [5,6]. In support of these observations, a recent study has shown the functional interaction between α4β2 nAChR and D_3_R [7].

In addition to their roles in nicotine addiction, the brain DAergic and nicotinic cholinergic systems are known to play important roles in the pathogenesis and management of the symptoms related to schizophrenia and Parkinson’s disease (PD). For example, dopamine and nicotinic receptors expressed in the prefrontal cortex are involved in the regulation of the negative symptoms of schizophrenia. In addition, L-DOPA-induced dyskinesiasn (LID), which is evoked through persistent stimulation of dopamine receptors, can be managed through activation of nicotinic receptors. This review focused on the interaction between the two neurotransmitter systems with respect to the pathogenesis and treatments of schizophrenia and PD.

### 1.1. DAergic Regulation of Motor Planning via Basal Ganglia

Control of motor function involves an intricate interplay between various brain regions, including the dorsolateral prefrontal cortex (DL-PFC), secondary motor cortex, primary motor cortex, and basal ganglia. The basal ganglia determine motor plans based on glutamatergic commands from the secondary motor cortex and DAergic input from the substantia nigra (SN).

**Figure 1:**
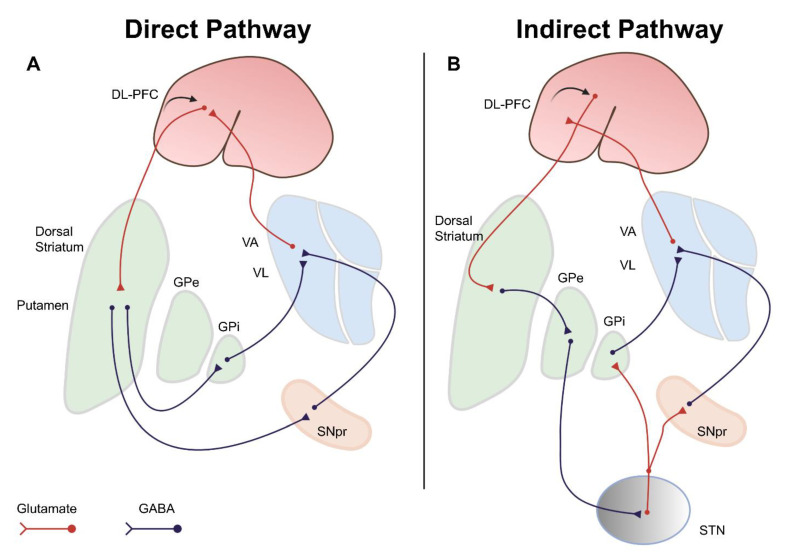
Direct and indirect pathways in the basal ganglia. Two principal pathways in the basal ganglia are involved in the selection of the motor plans. Excitatory pathways (glutamatergic) are shown in red, and inhibitory pathways (GABAergic) are shown in black. (**A**) The direct pathway employs the disinhibitory (GABA to GABA) pathway that provides signals from the putamen to the GPi/SNpr, leading to the activation of the thalamus that, in turn, stimulate the cortex. Through these connections, the direct pathway intensifies the motor plan prepared by the cortex. (**B**) In contrast, the indirect pathway employs the circuit composed of “inhibitory-stimulatory-inhibitory” connections to convert stimulatory corticostriatal glutamatergic input into inhibitory signals to the thalamus. The indirect pathway begins at the putamen, goes through the GPe, arrives at the STN, and then projects to the GPi/SNpr. The GPi/SNpr are connected to the thalamus, but the thalamic sites connected to the indirect pathway are different from the sites used by the direct pathway. Through these connections, the indirect pathway discourages motor plans other than those prepared by the cortex. GPi, globus pallidus internus; GPe, globus pallidus externus; SNpc, substantia nigra pars compacta; SNpr, substantia nigra pars reticulata; STN, subthalamic nucleus. Figures created with https://biorender.com/ (accessed on 4 March 2021).

As shown in Figure 1, the secondary motor cortex sends glutamatergic projections to basal ganglia. Basal ganglia use direct and indirect pathways that employ GABAergic medium spiny neurons (GABA-MSNs). GABA-MSNs originate from the putamen and regulate specific regions of the thalamus that return excitatory feedback to the cortex via glutamatergic neurons [8,9].

In the direct pathway (Figure 1A), the putamen gives rise to GABA-MSNs that project to the globus pallidus internus (GPi) and SN pars reticulata (SNpr). These inhibitory neurons, in turn, inhibit GABAergic neurons, which project from the GPi/SNpr to the ventral anterior (VA) and ventrolateral (VL) nucleus of the thalamus. These successive GABA neuronal connections functionally lead to disinhibition, resulting in the activation of specific regions in the VA/VL of the thalamus. Regions of the thalamus that are stimulated through the direct pathway project glutamatergic neurons to the specific regions of the supplementary motor area (SMA) to give permission for the motor plans prepared by the DL-PFC and SMA.

In the indirect pathway (Figure 1B), the putamen projects GABA-MSNs to the globus pallidus externus (GPe), and the GPe projects GABAergic neurons to the subthalamic nucleus (STN), resulting in the disinhibition of the STN. The STN projects glutamatergic neurons to GPi/SNpr, which, in turn, project GABAergic neurons to the VA/VL of the thalamus. Thus, the overall outcome of the indirect pathway is inhibitory. These GABAergic neurons from the GPi/SNpr innervate regions of the VA/VL that are not stimulated by the direct pathway, resulting in the selective activation of those regions that are responsible for transmitting the motor plans prepared by the cortex. Finally, the thalamic regions inhibited by the indirect pathway send attenuated glutamatergic signals to specific regions of the secondary motor cortex. This enables the so-called ‘double confirmation’, in which certain areas of the secondary motor cortex are selectively activated while nearby areas are suppressed. Thus, the medium spiny neurons (MSNs) in the direct pathway are considered the driving factor for movement facilitation under normal physiological conditions. In contrast, the MSNs of the indirect pathway are thought to be inhibited during performing purposeful movement but are more active during lack of normal movement. 

**Figure 2:**
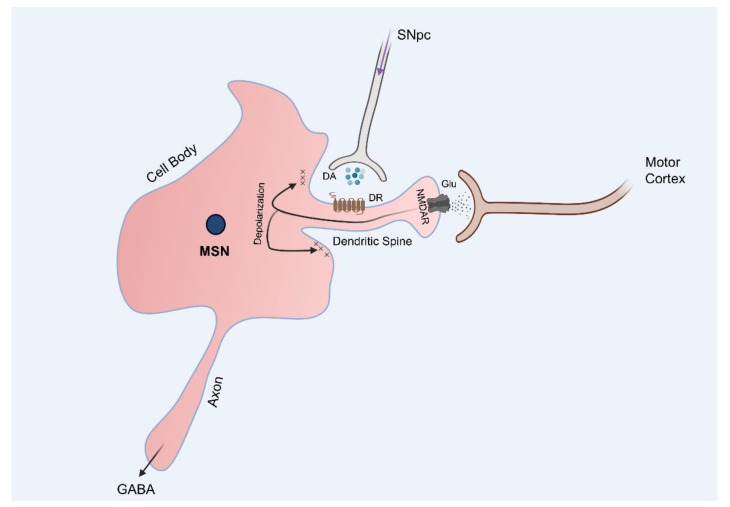
Roles of the nigrostriatal pathway in the regulation of the basal ganglia pathway. Medium spiny neurons (MSNs) in the putamen receive stimulatory input through the cortico-striatal glutamatergic neurons. They also receive dopaminergic input through nigrostriatal DAergic neurons. DA stimulates the direct pathway by acting on the D_1_Rs expressed on the MSNs that project to the GPi but inhibits the indirect pathway by acting on the D_2_Rs expressed on the MSNs that project to the GPe. Thus, degeneration of DAergic neurons originating from the SN results in an under-stimulation of the direct pathway and under-inhibition of the indirect pathway, resulting in problems with purposeful movement. DR, dopamine receptor; Glu, glutamine; NMDAR, NMDA receptor. Figures created with https://biorender.com/ (accessed on 13 April 2021).

GABA-MSNs synapse with striatal interneurons, as well as a number of incoming neurons, including corticostriatal glutamatergic and nigrostriatal DAergic neurons. The glutamatergic neurons projecting from the cortex synapse onto the dendritic spines of GABA-MSNs on which glutamate receptors are expressed. DA receptors are expressed on the neck of the synaptic buttons on which glutamate receptors are expressed. Nigrostriatal DA input to MSNs either increases or decreases glutamatergic signaling by acting on the D_1_ receptors (D_1_R) or D_2_ receptors (D_2_R) located on the direct and indirect GABA-MSNs, respectively (Figure 2) [8,10]. Overall, the direct pathway is stimulated but while the indirect pathway is inhibited, resulting in the facilitation of motor functions.

Cholinergic interneurons, another group of neurons that interact with GABA-MSNs, inhibit the direct pathway mainly through M4 receptors and stimulate the indirect pathway through M1 receptors. In PD, DAergic input to the putamen is decreased, and both the direct and indirect pathways are inhibited. This largely results in inhibition of intended movement and increased unintended movement at rest through the unopposed effects of cholinergic neurons [11]. As the balance between the direct and indirect pathways is required for the fine control of motor functions, degeneration of the DAergic nigrostriatal pathway results in abnormalities of movement, such as bradykinesia and resting tremors.

### 1.2. Brain DAergic System and DA Receptors

Four major DAergic pathways are found in the brain [12]. First is the mesolimbic tract pathway that projects from the VTA of the midbrain to the limbic system, including the NAc. Hyperactivity of this pathway is related to addiction and the positive symptoms of schizophrenia [13], and the antagonism of D_2_R in the mesolimbic pathway has been used to treat positive psychotic symptoms [14]. Second is the mesocortical tract that connects the VTA to the prefrontal cortex. Projections to the DL-PFC and ventromedial prefrontal cortex regulate cognition/executive functioning and emotions/affect, respectively [15]. A decrease in DAergic activity in the mesocortical projections to the DL-PFC is postulated to be responsible for the negative symptoms of schizophrenia [16]. In agreement with this, nicotine, which evokes DA release in the mesocortical pathway, is known to alleviate the negative symptoms. The third is the nigrostriatal tract, which connects the SN pars compacta (SNpc) and dorsal striatum, and is part of the extrapyramidal system and important in motor movement control. This pathway plays central roles in the pathogenesis of PD [17] and is also responsible for the extrapyramidal symptoms caused by typical antipsychotic drugs that antagonize D_2_Rs. Finally, the tuberoinfundibular pathway refers to the DAergic neurons that project from the hypothalamus to the anterior pituitary (infundibular region). DA released from the pituitary blocks the secretion of prolactin [18]; thus, blockade of D_2_Rs in this pathway can lead to hyperprolactinemia, which clinically manifests as amenorrhea, galactorrhea, and sexual dysfunction.

Based on their pharmacological and functional characteristics, DA receptors are largely classified into D1- and D2-like receptors [19,20], which positively and negatively regulate adenylyl cyclase, respectively. When they are classified according to the genes encoding them, the D1-like receptors are subdivided into the D_1_Rs and D_5_ receptors (D_5_R) [21,22], whereas the D_2_, D_3_, and D_4_ receptors constitute the D2-like receptors (D_2_R, D_3_R, D_4_R) [23,24,25].

DA receptors have different affinities to DA; D_3_R has the highest, followed by D_5_R, D_4_R, D_2_R, and D_1_R [26]. Thus, it is possible that DA activates different subtypes of DA receptors depending on its concentration in the biophase [27]. In addition to their affinity to DA, the DA receptor expression levels within particular brain circuits or regions play key roles in determining their physiological functions [28].

DA receptors in the prefrontal cortex, striatal GABAergic MSNs and cholinergic interneurons, and nucleus accumbens are closely related to schizophrenia, drug addiction, and PD. DAergic neurons in the VTA and SN [29], which provide DAergic inputs to the striatum and prefrontal cortex, are spontaneously active with firing patterns that range from tonic to phasic [30,31]. Because of their differences in affinity to DA, tonic and phasic patterns of DA release differently influence the activities of certain neuronal pathways in which more than one DA receptor subtype is expressed. For example, when large amounts of DA are released during phasic firing, D_1_Rs can be activated [32], but D_2_Rs respond to a broader range of stimuli [33].

The D_1_R, which is highly expressed on dendritic spines in layer 3 of the DL-PFC [34,35], has been implicated in the control of working memory [36,37], and working memory dysfunction is a prominent feature of schizophrenia [38]. Expression of D_1_R is reduced in the prefrontal cortex of schizophrenics, and this reduction is related to the severity of negative symptoms, such as emotional withdrawal [39,40].

### 1.3. Brain Cholinergic System and Nicotinic Receptors

Brain cholinergic projections belong to one of two systems depending on where the cell bodies are located: the magnocellular basal forebrain cholinergic system or the brain stem cholinergic system [41,42].

Cell bodies are located in the medial septal nucleus, diagonal band of Broca, and nucleus basalis of Meynert in the basal forebrain cholinergic system, and they send cholinergic axons to the neocortex, as well as limbic cortices [41]. The basal forebrain cholinergic system plays critical roles in several aspects of cognition, including attention, cognition, working memory, spatial learning, and associative learning [43].

The brainstem cholinergic system sends projections from the pedunculopontine tegmental nucleus and laterodorsal pontine tegmentum to various brain regions that include the basal ganglia, midbrain, cerebellum, and thalamus [41,44,45]. Studies show these cholinergic systems to be involved in controlling the sleep-wake cycle and positive reinforcement [46,47].

nAChRs are members of the superfamily of Cys-loop ligand-gated ion channels [48]. All nAChRs are composed of pentameric subunits arranged around a central ion pore through which cations pass. nAChRs are classified into different subfamilies based on several criteria that include subunit composition, sensitivity to different ligands, and regulatory properties, such as desensitization. Members of subfamilies I (α9-α10) and IV (muscle nAChR subunits), and one member of subfamily II, α8, are not expressed in mammalian brain [49,50]. Thus, in this review, we discussed the roles of brain nicotinic receptors focusing on subfamily III (α2-α6, β2-β4) and α7 nAChRs.

Different nAChR subtypes show different kinetics of activation and desensitization. α7* nAChRs, which have a lower affinity to nicotine, are rapidly activated and desensitized by high doses of nicotine [51,52]. At physiological concentrations, however, only a small proportion of the α7* nAChR population is occupied by nicotine and desensitized. Owing to their rapid kinetics, α7* nAChRs rapidly recover from desensitization when nicotine unbinds. Consequently, only a very small portion of the α7 population is desensitized under physiological conditions and the majority of the receptors are available for activation. In contrast, β2* subtypes—the predominant nAChR subtype on midbrain DAergic and GABAergic neurons—have a higher affinity for nicotine and undergo strong desensitization [53]. It has been reported that α4β2 nAChRs containing α5 subunits recover more rapidly from desensitization [54].

Molecular biological manipulation, such as gene knockout, has helped us to understand the functional roles of specific nAChR subtypes. For example, β2 gene knockout has revealed the essential roles of 4β2- and α6β2-containing receptors for nicotine reward and addiction [55,56,57]. In contrast, α7 nAChRs are known to have opposite roles in tobacco dependence [58]. Thus, inhibition of α6β2* nAChRs and stimulation of α7 nAChRs, in connection with the supportive roles of PPARα, has been suggested as a strategy for tobacco cessation [59,60].

### 1.4. Functional Interaction between the Cholinergic and DAergic Systems

It has been reported that systemic administration of nicotine causes DA release in the NAc [61] by acting on the specific nAChR subtypes expressed on the cell body and terminals of the DA neurons that project from the VTA to the NAc [62,63]. Among a number of nAChR subtypes, those containing β2 and/or α6 subunits are known to play critical roles in regulating DA-mediated neurotransmission in the mesolimbic pathway. Many of the studies on nicotine addiction/dependence have focused on the α4β2 receptor subtype, which is ubiquitously expressed throughout the brain. More recently, α6β3 receptors—the more selectively expressed in DAergic neurons [64,65]—have been shown to be the primary receptors on DAergic neuronal terminals that stimulate DA release [66,67].

In addition to stimulating DA release, nicotine is also known to promote the survival of nigrostriatal DA neurons, supporting the beneficial effects of smoking on PD development [57,68]. In agreement with these results, nicotine is known to be involved in the morphological remodeling of DAergic neurons and regulates various genes that regulate neuronal morphogenesis [69,70].

Some of nAChR subtypes colocalize with D2-like receptors in DAergic nerve terminals and it has been reported that DA receptors are needed for nicotine to exert its actions on DAergic neurons [67,71]. For example, α4β2 nAChRs colocalize with D_3_Rs in the somatodendritic area of DAergic neurons [72], and nicotine increases D_3_R expression in the shell of the nucleus accumbens [73]. In addition, nicotine increases dendritic arborization of cultured DAergic neurons, and this effect is either blocked by D_3_R antagonists or disappears in D_3_R knockout mice [70].

## 2. Roles of the DAergic and Nicotinic Cholinergic Systems in the Pathogenesis of Schizophrenia

Approximately 1% of the general population is schizophrenic, showing an approximately 50% concordance rate. Symptoms of schizophrenia can be categorized into three groups: positive, negative, and cognitive [74]. Positive symptoms are characterized by hallucinations and delusion. Patients lose their sense of reality and often create their own reality. Negative/deficit symptoms accompany motional flattening, anhedonia, withdrawal from social interaction, loss of motivation, poverty in speech and thought, and patients lose their normal emotional and behavioral capacities. Cognitive/disorganization symptoms are believed to occur when the patient’s memory and sometimes movement are hampered. Symptoms include working memory and execution problems, disorganized speech, loosening of associations, neologisms, blocking, and clanging [75].

### 2.1. Roles of the DAergic System in Schizophrenia

**Figure 3:**
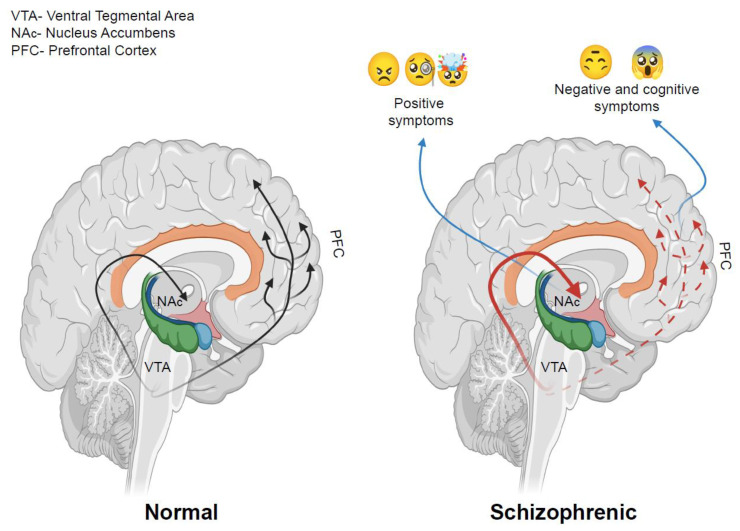
Roles of two different brain dopaminergic pathways in schizophrenia. Over-activation of the mesolimbic DAergic system is believed to correlate with the positive symptoms of schizophrenia. Hypofunctioning of the mesocortical DAergic pathway, which is associated with cognitive function, e.g., working memory, is believed to correlate with the negative symptoms of schizophrenia. NAc, nucleus accumbens; PFC, prefrontal cortex; VTA, ventral tegmental area. Figures created with https://biorender.com/ (accessed on 12 April 2021).

The pathogenesis of schizophrenia has previously been most commonly explained by the ‘DA hypothesis’. This hypothesis was reverse postulated based on the observation that DA-increasing drugs, such as amphetamines and cocaine, can cause psychotic symptoms, while antipsychotic drugs that antagonize D_2_Rs reduce psychotic symptoms [76,77]. However, subsequent studies have shown that multiple neurotransmitter systems other than the DAergic system are also involved in the pathogenesis of schizophrenia. Decreased rather than increased DAergic activity in the prefrontal cortex is responsible for some of the cognitive and negative symptoms of schizophrenia (Figure 3), and the newer class of ‘atypical antipsychotics’ are less potent antagonists of D_2_Rs compared to the first generation of typical antipsychotics, in that they act on other targets, such as the 5-HT_2A_ receptor [78].

The relationship between the hyperactive mesolimbic DAergic pathway and the positive symptoms of schizophrenia is relatively well established. In contrast, the negative symptoms are more difficult to understand and might be explained in part by the so-called ‘hypoactive mesocortical’ pathway. For example, many of these cognitive deficits are explained by dysfunction of the DL-PFC [34]. Accordingly, the brains of schizophrenic patients show evidence of dendritic atrophy of pyramidal cells in the DL-PFC, reductions in cortical DA content, and possible rebound increases in D_1_Rs [40].

### 2.2. Functional Interaction between the DAergic and Nicotinic Cholinergic Systems in the Pathogenesis and Treatment of Schizophrenia

As discussed above, the positive and negative symptoms of schizophrenia have been explained by hyperactive mesolimbic and hypoactive mesocortical DAergic pathways, respectively, and the blockade of mesolimbic D_2_Rs and enhancement of mesocortical D_1_Rs have beneficiary effects on positive and negative symptoms [79,80]. This so-called, ‘DA hypothesis’ of schizophrenia can be further refined when the roles of brain nAChRs, NMDA receptors (NMDAR), and GABA receptors are also considered [81].

Nicotine is known to activate DAergic neurons to release DA both in the VTA and cortex [53,82], suggesting that nicotine could affect both positive and negative symptoms [83,84]. Post-mortem studies from schizophrenic patients have revealed a disturbance in nAChR expression, in particular, α7 and α4β2 subunits [85,86]. It is believed that the positive symptoms of schizophrenia are related to D_2_R and β* (α4β2 and α6β2) nAChRs, but D_1_R and α7 nAChR are related to the negative symptoms [87,88,89].

In addition to the roles of the DAergic and nicotinic cholinergic systems in the pathogenesis of schizophrenia, a glutamate hypothesis has been also proposed [90] based on the observations that NMDAR antagonists induce symptoms similar to those of schizophrenia [91,92]. According to this hypothesis, the hypofunctioning of NMDARs on parvalbumin-positive GABA interneurons within the prefrontal cortex is important in the pathogenesis of schizophrenia, resulting in impairment of lateral inhibitions of pyramidal cells in the DL-PFC [93,94]. Further, post-mortem studies of schizophrenic patients have shown reduction or alterations in the expression levels and trafficking of NMDARs [95,96].

**Figure 4:**
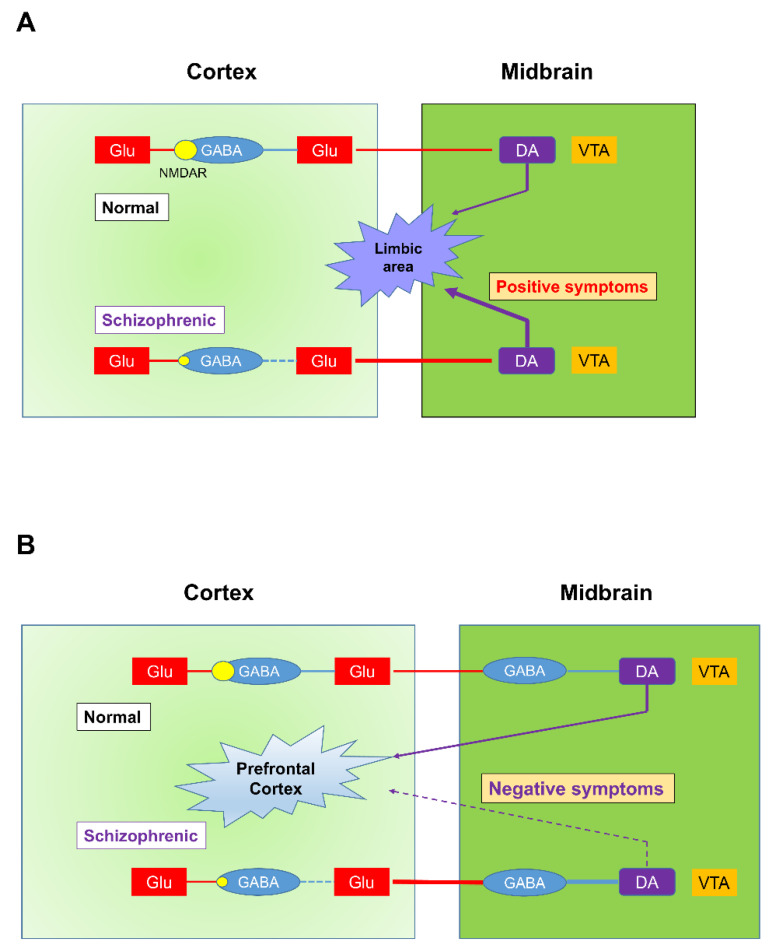
Differential involvement of multiple signaling systems in the manifestation of positive and negative symptoms of schizophrenia. According to recent studies on the mechanistic hypothesis of schizophrenia, signaling systems other than the DAergic system also play important roles. For example, abnormalities in the glutamatergic or GABAergic system could be involved in the dysregulation of the DAergic system, which is believed to be the final common pathway in schizophrenia. (**A**) Glutamate hypofunctioning hypothesis for the positive symptoms of schizophrenia. (Upper) Normal Glu-GABA-Glu neuronal circuitry with respect to mesolimbic DAergic projection. A pyramidal Glutamate neuron (the first neuron) synapses on a GABA interneuron (the second neuron), which next inhibits a secondary cortico-tegmental pyramidal glutamatergic neuron (the third neuron). These series of connections lead to the firing of mesolimbic dopaminergic neurons at the normal rate. (Lower) Abnormal Glu-GABA-Glu-DA neuronal circuitry. The NMDAR on the GABAergic neuron is suboptimal, and the second glutamatergic neuron is not inhibited properly, resulting in overactivation of the mesolimbic DAergic system that projects to limbic area. (**B**) Glutamate hypofunctioning hypothesis for the negative symptoms of schizophrenia. (Upper) Normal Glu-GABA-Glu-GABA neuronal circuitry with respect to the mesocortical DAergic pathway. In this pathway, another GABAergic connection is inserted between the cortico-tegmental glutamatergic and mesocortical DAergic neurons at the level of the brainstem. (Lower) In schizophrenia, a loss of NMDA activity on the first GABAergic neuron leads to consecutive activation of subsequent Glu-GABA neurons, which results in the inhibition of DAergic neurons located in the VTA. The decrease in DA input to the frontal cortex correlates with the negative symptoms of schizophrenia. Adapted from Reference [81].

Indeed, the DA hypothesis of schizophrenia can better explain both the positive and negative symptoms when the roles of NMDARs are considered in the neuronal circuitry involved in schizophrenia [81,97]. For instance, the positive and negative symptoms of schizophrenia can be explained by the Glu-GABA-Glu-DA and Glu-GABA-Glu-GABA-DA neuronal circuitry that leads to DAergic projection to the NAc and prefrontal cortex, respectively [81] (Figure 4).

In the cortex-VTA-NAc circuit that is related to the positive symptoms of schizophrenia, glutamatergic neurons project axons to the GABAergic interneurons in the cortex, which, in turn, synapse on the secondary cortical pyramidal glutamatergic neurons that project to DAergic neurons in the VTA. DAergic neurons, therefore, project to the nucleus accumbens, completing the ‘Glu-GABA-Glu-DA’ circuitry. When this circuit is disturbed by the hypofunctioning of NMDARs on the GABAergic neurons, secondary glutamatergic neurons will be overly stimulated, leading to the hyperactivation of DAergic neurons that project to the NAc [98].

In the cortical-VTA-cortical circuit that is related to the negative symptoms of schizophrenia, secondary GABAergic midbrain interneurons are additionally inserted into the middle of the circuit. In this circuit, the secondary glutamatergic neurons synapse on the GABAergic interneurons in the midbrain, which, in turn, synapse on the VTA-DAergic neurons that project back to the frontal cortex (Glu-GABA-Glu-GABA-DA). The hypofunctioning of the NMDARs on the primary GABAergic neurons results in hypoactivity of the mesocortical DAergic pathway and inadequate supply of DAergic inputs to the prefrontal cortex, causing negative symptoms.

Unlike positive symptoms wherein the mesolimbic DAergic pathway plays a key role, various brain regions and multiple receptors are likely to be involved in the development of negative symptoms. For example, the prefrontal cortex, thalamus D_1_Rs, NMDARs, and α7 nAChRs are known to be involved in the development of negative symptoms. This supports the previous notion that cognitive deficits in schizophrenia might be associated with reduced DAergic input to the PFC [99,100].

The thalamic mediodorsal (MD) nucleus plays a key role in the communication between distinct associative cortical areas [101]. Axons derived from the thalamic MD nucleus synapse onto the dendrites of the DL-PFC pyramidal neurons that are responsible for executive control and working memory. In schizophrenia, neurons of the thalamic MD are known to degenerate, reducing the size of this area [102]. The use of dendrites located in the DL-PFC reduces with the decreasing number of nerves entering from the MD nucleus, leading to selective atrophy of the pyramidal cell microcircuits in deep layer III of the DL-PFC, as well as compensatory weakening of related GABAergic interneurons.

Glutamatergic pyramidal cells form synapses between axon terminals and dendritic spines in layer III of the DL-PFC. NMDARs and α7 nAChRs are co-expressed in the postsynaptic membrane of the layer III spines where D_1_Rs are concentrated. Moderate levels of D_1_R stimulation in the DL-PFC are known to be essential for optimal working memory function [103]. D_1_Rs are often co-localized with hyperpolarization-activated cyclic nucleotide gated 1 (HCN1) channels on spines [40], and D_1_R-mediated increases in cAMP are expected to open HCN channels. Because HCN channels mediate rhythmic depolarization of the membrane potential when cells are hyperpolarized [104], activation of D_1_Rs is expected to raise the membrane potential near to threshold, evoking more frequent action potentials. Therefore, it is believed that HCN channels are responsible for filtering out low-frequency inputs, thereby improving the selectivity for synchronous synaptic inputs [105,106]. Synchronization between inputs from glutamate axons and the activation of HCN channels though D_1_Rs could, thus, be an important functional device for optimal synaptic transmission between pyramidal cells on the DL-PFC. To this end, DA that elevates intracellular cAMP by acting on D_1_Rs has an inverted U influence on the firing of the delay cells and cognitive performance, where moderate levels are essential for functioning, but excessive stimulation suppresses neuronal firing and impairs cognitive abilities [107].

**Figure 5:**
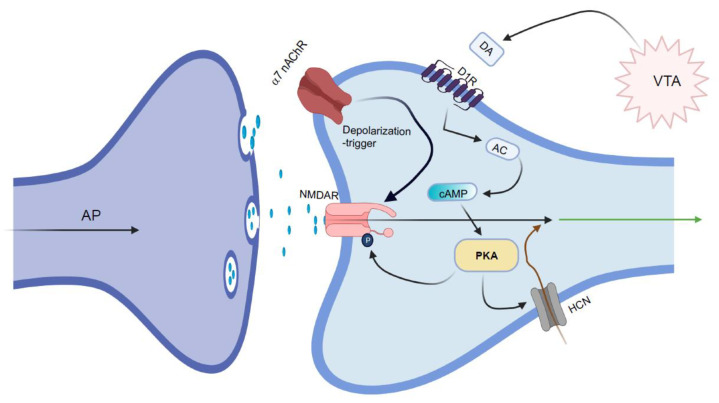
Schematic illustration of the synaptic connection between glutamatergic pyramidal cells in layer III of the dorsolateral prefrontal cortex. Glutamatergic pyramidal cells form synapses between axon terminals and dendritic spines. NMDARs expressed on the postsynaptic membrane are triggered by the stimulation of α7 nAChRs that are co-expressed in postsynaptic cells. D_1_Rs are also expressed on postsynaptic dendritic spines, where they often co-localize with hyperpolarization-activated cyclic nucleotide (HCN) channels. HCN channels are activated by cAMP-PKA signaling, leading to rhythmic depolarization of the cells. There is optimism that synaptic transmission might be possible when signaling NMDAR is harmonized with stimulatory effects via D_1_R. Adapted from Reference [97]. AC, adenylyl cyclase; AP, action potential. Figures created with https://biorender.com/ (accessed on 15 April 2021).

Overall, NMDARs, α7 nAChRs, and D_1_Rs play critical roles in synaptic transmission between glutamatergic axonal terminals and dendritic spines on layer III pyramidal cells. This synaptic transmission allows the mutual excitation of glutamatergic neurons (Figure 5). Unlike conventional glutamate synapses, where AMPA receptors trigger NMDAR channel opening, the glutamate synapses between pyramidal cells use cholinergic stimulation of α7 nAChRs to provide the depolarization required to open NMDARs [108]. Indeed, defects in both NMDARs and α7 nAChRs are known to be linked to schizophrenia.

Based on these promising experimental results, the α7 nAChR has been proposed as a potential target for managing schizophrenia [85,109], and α7-selective agonists and allosteric modulators have been tested in small-scale trials for the treatment of schizophrenia [110,111]. 3-(2,4-dimethoxybenzylidene)-anabaseine (DMXB-A) is one example of these [112], and several more compounds have shown promising results in early clinical studies [109].

## 3. Roles of the Interaction between the DAergic and Nicotinic Cholinergic Systems in the Pathogenesis and Treatment of PD

PD is characterized by movement disabilities, including tremors, rigidity, bradykinesia/hypokinesia, and postural instability, as well as numerous other deficits affecting cognition, sleep, and autonomic nervous system function [113,114]. PD is associated with a generalized loss of neurons throughout the brain, with the most prominent feature being the degeneration of nigrostriatal DAergic neurons.

PD is the second most common neurodegenerative disease after Alzheimer’s disease, affecting approximately 1–2% of the population in the USA. Although most cases of PD occur sporadically, approximately 5–10% are inherited (familial PD). Recent genomic analyses using samples obtained from patients with familial PD have mapped many PD-related (PARK) loci and identified several putative genes, including leucine-rich repeat kinase 2 (LRRK-2), PARK2 (parkin), DJ-1, and PTEN-induced kinase 1 (PINK1).

Mutations in LRRK2 have various effects, including disruption of the endosome and lysosome degradation pathways, leading to increased concentrations of α-synuclein that facilitate its aggregation, resulting in the formation of Lewy bodies (LB). Mutations in LRRK2 also alter specific signaling pathways, such as the Ras and MAPK pathways that support vesicular transport along axons and DA release, and increase the tau protein phosphorylation and accumulation [115] to cause an abundance of neurofibrillary tangles. These three effects together result in DAergic neuronal death [116,117]. Mutations in PARK2, which encodes an E3 ubiquitin ligase [118], inhibit proteasomal degradation of α-synuclein, resulting in LB formation and DAergic neuronal cell death [119,120]. Further, DJ-1, a redox-sensitive chaperone and sensor for oxidative stress, inhibits the aggregation of α-synuclein, and DJ-1 mutations lead to the inhibition of proteins related to anti-oxidation and mitochondrial functions, resulting in an increase in reactive oxygen species (ROS) and mitochondrial dysfunction, and destruction of DAergic neurons [121,122]. PINK1, a mitochondrial serine/threonine-protein kinase, is closely associated with mitochondrial quality control by identifying damaged mitochondria and targeting them for degradation [123,124]. Mutations in PINK1 exert similar effects as those reported in DJ-1 mutations, and tau protein phosphorylation is increased.

Various genetic and environmental factors converge into four fundamental mechanisms; oxidative stress, mitochondrial complex I dysfunction, impairment of the ubiquitin-protease pathway, and accumulation and aggregation of misfolded or unfolded proteins.

### 3.1. PD and the DAergic System

A number of studies have shown the involvement of DA receptor subtypes in PD. Studies in D_1_R knockout mice have shown that D_1_R and D_2_R are segregated on striatal projection neurons, with D_1_R regulating the direct striatal output pathway. D_1_R knockout mice mostly show increased locomotor hyperactivity or a decrease in spontaneous exploratory activities, as determined by a decrease in rearing behavior [125,126]. Overall, these studies suggest that D_1_Rs regulate the neurochemical architecture of the striatum and are critical for the expression of normal motor activity [125].

Studies using knockout mice have shown that the D2-like receptor family (mainly D_2_R) plays a more direct role in PD. A long isoform of an alternatively spliced variant of D_2_R, D_2L_R, acts mainly at postsynaptic sites and is suggested to be related to the extrapyramidal side effects of typical antipsychotics [127,128]. In addition, it has been suggested that the SNpc of D_2_R knockout mice contain more LB-like cytoplasmic inclusions containing α-synuclein, compared to wild-type mice [129].

The severity of PD is reportedly related to a decrease in D_3_Rs in the brain [130] and these receptors could be potential biomarkers for PD [131]. In addition, D_3_R agonists can decrease cellular accumulation of α-synuclein, enhance BDNF secretion, decrease neuroinflammation, and improve motivational deficits [132,133].

D_4_R knockout mice show significantly less exploratory behavior and rearing activity than wild-type mice [134,135], and increased avoidance behavior to unconditioned stimuli [136]. These results indicate that D_4_R could also play a vital role in impulse control disorder, which is one of the major symptoms in PD [137].

Overall, D2-like receptors appear to play major roles in PD, although D1-like receptors probably have indirect roles.

### 3.2. Roles of the Cholinergic System in PD

The balance between the DAergic and cholinergic systems in the striatum is critical for the proper regulation of motor functions. The striatum contains several neuronal types that include projecting GABA-MSNs (~95%), medium-size striatal GABAergic interneurons, and large aspiny cholinergic interneurons (CINs) [138,139]. Most striatal cholinergic innervations are provided by CINs [140], which comprise less than 3% of the cells in the striatum. Morphologically, CINs can be easily distinguished from other striatal neurons as they are significantly larger than other neurons (approximately 20–50 μm in diameter) and possess extensive axonal and dendritic arborizations [141,142].

**Figure 6:**
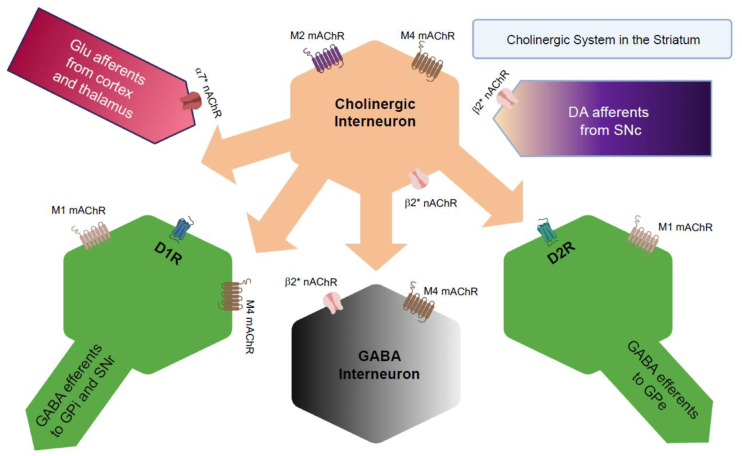
Characterization of cholinergic interneurons in the striatum. Schematic representation of interconnections between CINs and other neurons in the striatum. The direct MSNs that project preferentially to the GPi/SNpr express D_1_Rs along with M1 (M1R) and M4 receptors (M4R). The indirect MSNs that project to the GPe express D_2_R along with M1Rs. β2* nAChs and α7 nAChRs are expressed on the axonal terminals of the nigrostriatal DAergic neurons and corticostriatal glutamatergic neurons. CINs also express multiple cholinergic receptors that include β2* nAChRs, M2Rs, and M4Rs. Figures created with https://biorender.com/ (accessed on 12 April 2021).

Owing to their morphological features, CINs make widespread synaptic connections with other neurons, including nigrostriatal DAergic and corticostriatal glutamatergic afferents, serotonergic afferents from the dorsal raphe, and GABAergic efferent and interneurons [143] (Figure 6). ACh release from CINs is controlled by various neurotransmitters that act on a multitude of receptors expressed on CINs, for example, GABA_A_ receptors, metabotropic glutamate receptors, ionotropic glutamate receptors, and DA receptors [144,145,146,147,148].

CINs are tonically active and continuously release ACh in a pulsatile manner. The ACh released from CINs subsequently acts on ACh receptors located on neuronal terminals and/or cell bodies in the striatum to modulate neurotransmitter release from DAergic and cortical glutamatergic neurons [138,149].

The neuronal terminals of nigrostriatal DAergic projections heavily overlap with CINs in the striatum [139,150] and mutually regulate the release of DA and ACh from each neurons. For example, DA released from nigrostriatal neurons exerts stimulatory and inhibitory influences on ACh release by acting on D_1_Rs and D_2_Rs, respectively [147,148], allowing for the fine-tuning of DA receptor-mediated regulation of locomotor activity [151].

ACh released from striatal CINs also regulates DA release from the striatum by acting on the nAChR subtypes expressed on different neuronal terminals. ACh stimulates DA release by directly acting on α4β2* and α6β2* (α4β2, α4α5β2, α6β2β3, α4α6β2β3) subtypes expressed on DAergic neurons [152,153]. The majority of nAChR subtypes on DAergic neurons (β2*) are activated by nicotine and then desensitized, allowing the excitation of DAergic neurons for a short period of time before they are desensitized. Striatal α7 nAChRs located on corticostriatal glutamatergic efferents indirectly regulate DA release by modulating striatal glutamate release [154]. In contrast to most of the β2* nAChR subtypes, α7 nAChRs are not significantly desensitized by the concentrations of nicotine obtained from smoking.

### 3.3. Functional Interaction between DAergic and Nicotinic Cholinergic Systems in the Pathogenesis and Treatment of PD

Nicotine is known to reduce nigrostriatal-damaging processes [155,156], and a number of epidemiological studies have shown that the incidence of PD is reduced by approximately 50% in smokers [157,158]. Nicotine is also known to reduce LID [155], one of the most serious side effects caused by the chronic use of L-DOPA. As suggested in a recent review [159], the connection between the reduced incidence of PD and tobacco use is probable because the protective effects occur in a dose- and time-dependent manner, and has also been confirmed in twin studies [160].

The nicotinic and DAergic systems are closely associated in normal physiology and the pathogenesis of PD, which could be due to the extensive overlap between the two systems. For example, both striatal nAChR and DA receptor subtypes decrease with striatal DAergic degeneration [161], which is the most prominent feature of PD. Loss of nAChR subtypes occurs in the order of α6α4β2β3 > α6β2β3 > α4β2 [69,152]. α7 nAChRs are not affected by nigrostriatal damage probably because they are not expressed on DAergic terminals [72,162]. DA release and the stimulation of D_1_Rs and D_2_Rs on GABA-MSNs are also reduced, leading to an overall decline in movement facilitation mediated by the direct pathway. This results in the bradykinesia, rigidity, freezing, and other motor deficits observed in PD.

L-DOPA is one of the most effective therapies for PD; however, chronic use of L-DOPA results in various adverse effects, such as neuropsychiatric problems and the development of abnormal involuntary hyperkinetic movements (dyskinesias). Currently, amantadine [163], levetiracetam, an antiepileptic [164], and nicotine [155,165] have been shown to improve LID.

The nigrostriatal DAergic system has been shown to be a key player in the development of LID [166,167]. Administration of L-DOPA is thought to lead to unregulated DA release and excessive activation of the striatal GABA-MSNs expressing D_1_Rs (direct pathway). The GABA-MSNs expressing D_2_Rs (indirect pathway) also become overactive via D_2_R-mediated inhibition of the inherent inhibitory nature of the indirect pathway, which leads to the overall enhanced motor activity, a characteristic of dyskinesias [168,169].

As expected from their roles in the regulation of the nigrostriatal DAergic system, striatal CINs and nAChRs have also been indicated in the pathogenesis of LID. For example, studies in nAChR null mutant mice showed that the β2* and α7 subtypes are related to LID [170,171,172]. In addition, ablation or long-term stimulation of CINs, which results in nAChR desensitization and a consequent functional receptor blockade [173,174], effectively prevent LID without affecting the therapeutic efficacy of L-DOPA [175,176].

Receptor desensitization may subsequently lead to further molecular changes that mediate overall functional changes. Nicotine is known to modulate neurotoxicity by enhancing phosphatidylinositol 3-kinase and altering levels of phosphorylated Akt, as well as Src, B-cell lymphoma (Bcl) 2, and Bcl-x [177,178]. The MAPK/ERK and JAK2/STAT3 pathways have also been implicated in nAChR-mediated neuroprotection [179,180]. In addition, other downstream mechanisms, including alterations in phospholipase C [181], nerve growth factor [182], proinflammatory cytokines [183], caspases, and reactive oxygen species [184], could be involved.

Overall, these results demonstrate that striatal CINs play a critical role in LID by modulating the nigrostriatal DAergic pathway. Nicotine and nAChR drugs targeting β2* and α7 nAChRs could be useful strategies to counteract LID.

## 4. General Mechanism of Nicotine-Induced Neuronal Protection

### 4.1. Nicotine-Induced Changes in the Microenvironment of DAergic Neurons

Macrophages, represented by microglial cells in the brain, are innate immune cells that serve as the first line of defense against invading pathogens. Under normal conditions, they maintain the M0 phenotype, which can be activated and converted into two different phenotypes: M1, a pro-inflammatory macrophage, and M2, an anti-inflammatory macrophage [185,186].

**Figure 7:**
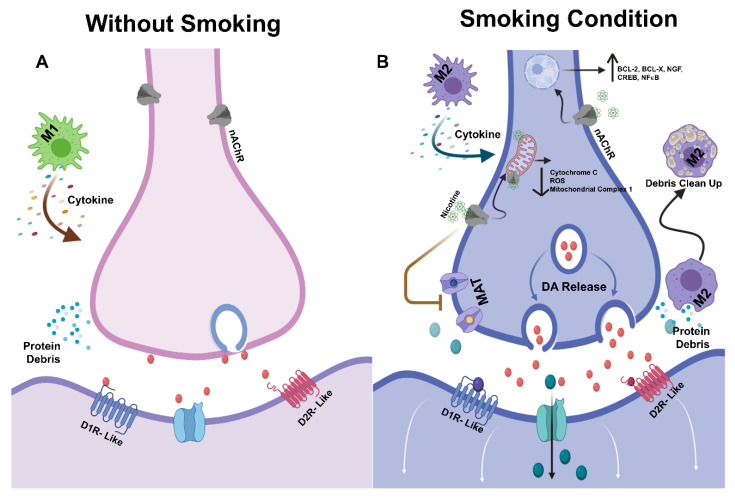
General mechanism of nicotine-induced neuronal protection. (**A**) Under degenerative inflammatory conditions, resident M0 macrophages polarize into M1 macrophages or pro-inflammatory macrophages, which release inflammatory cytokines. Cellular debris also deteriorates the cellular conditions of DAergic neurons. (**B**) Nicotine provides protection to injured DAergic neurons. Cellular protection can occur either via improvement of the neuronal microenvironment or by direct effects on DAergic neurons. Nicotine either converts resident M0 macrophages into M2 anti-inflammatory macrophages or it upregulates anti-apoptotic genes by acting on nAChRs. Figures created with biorender.com/ (accessed on 21 February 2021).

In the case of neurodegenerative diseases, such as PD, macrophages are usually converted into the M1 phenotype and cause local inflammation (Figure 7A), as well as neuronal death [187,188]. In contrast, nicotine is known to convert the M0 phenotype into the M2 phenotype, which prevents neuronal inflammation, as well as cell death (Figure 7B) [189,190].

Cytokine storm is one of the leading causes of neuronal cell death. Pro-inflammatory cytokines are usually released by M1 macrophages that predominate in neurodegenerative conditions (Figure 7A) [191,192]. Nicotine, by converting macrophages into the M2 phenotype that release anti-inflammatory cytokines, prevents inflammatory tissue injury and DAergic neuronal death [191,193]. In addition, the M2 phenotype is known to prevent DAergic neuronal death by cleaning up the accumulated toxic cellular debris (Figure 7B) [194].

### 4.2. Direct Effect of Nicotine on DAergic Neuronal Cell Survival

In addition to improving microenvironments, nicotine is known to have a beneficial effect on the survival of DAergic neurons, for example, by upregulating proteins responsible for neuronal survival, decreasing endogenous DA metabolism, and protecting mitochondria.

First, stimulation of the nAChRs expressed on DAergic neurons is known to upregulate the expression of several anti-apoptotic proteins, such as BCL-2, BCL-X, CREB, BDNF, NFκB, and NGF. These proteins play important roles both in neuronal survival and proliferation (Figure 7B) [195,196,197].

Second, 2, 3, 6-trimethyl-1, 4-napthoquinone (TMN), one of the components of cigarettes, is renowned for monoamine oxidase inhibitor. Thus, as a result of cigarette consumption, cellular levels of DA are increased along with a decrease in oxidative stress, which are beneficial for PD patients [198,199]. In addition, nicotine, the major component of cigarettes, improves the symptoms of PD patients by increasing DA release from DAergic neurons (Figure 7B) [66,151].

Third, damage to mitochondria is one of the major causes of DAergic neuronal death [200,201]. Unlike ACh, which is too hydrophilic to cross the cell membrane, nicotine can easily cross the cell membrane. Thus, it is possible that nicotine activates the nAChRs attached to internal organoid membranes, such as those of mitochondria [202,203]. By acting on mitochondrial nAChRs, nicotine produces antioxidant effects by modulating generation of ROS. In addition, nicotine prevents mitochondrial swelling and cytochrome c release independent of nAChRs, and reduces leakage of electrons during transport (Figure 7B) [198,204,205,206].

## 5. Conclusions

The DAergic and nicotinic systems of the brain play critical roles in nicotine addiction, schizophrenia, and PD. In considering the two systems together, it is possible to hypothesize mechanisms for these diseases that have not been previously considered. In particular, the interaction between these two systems allows for novel therapeutic approaches to treatments for related diseases, with fewer side effects. For example, understanding the regulatory roles of α7 nAChRs on pyramidal cells in the DL-PFC could support a mechanistic approach to treating the negative symptoms of schizophrenia and provide new therapeutic directions. Furthermore, elucidation of the critical role that α7 nAChRs play in the treatment of LID is enabling the introduction of novel therapeutic agents. Finally, in order to have more fundamental understanding behind various roles of nAChRs, it may be necessary to reconsider the nature and characteristics of nAChRs. For example, a recent study [207] has shown that α4β2 nAChR employs a metabotropic signaling pathway similar to those of GPCRs.

## Data Availability

Data sharing not applicable.

## References

[1] Di Chiara G. (2000). Role of dopamine in the behavioural actions of nicotine related to addiction. Eur. J. Pharm..

[2] Pidoplichko V.I., Noguchi J., Areola O.O., Liang Y., Peterson J., Zhang T., Dani J.A. (2004). Nicotinic cholinergic synaptic mechanisms in the ventral tegmental area contribute to nicotine addiction. Learn. Mem..

[3] Sarter M. (2015). Behavioral-Cognitive Targets for Cholinergic Enhancement. Curr. Opin. Behav. Sci..

[4] Wu J., Gao M., Shen J.X., Shi W.X., Oster A.M., Gutkin B.S. (2013). Cortical control of VTA function and influence on nicotine reward. Biochem. Pharm..

[5] Le Foll B., Schwartz J.C., Sokoloff P. (2003). Disruption of nicotine conditioning by dopamine D(3) receptor ligands. Mol. Psychiatry.

[6] Le Foll B., Collo G., Rabiner E.A., Boileau I., Merlo Pich E., Sokoloff P. (2014). Dopamine D3 receptor ligands for drug addiction treatment: Update on recent findings. Prog. Brain Res..

[7] Acharya S., Kim K.M. (2019). alpha4beta2 nicotinic acetylcholine receptor downregulates D3 dopamine receptor expression through protein kinase C activation. Biochem. Biophys. Res. Commun..

[8] Albin R.L., Young A.B., Penney J.B. (1989). The functional anatomy of basal ganglia disorders. Trends Neurosci..

[9] DeLong M.R. (1990). Primate models of movement disorders of basal ganglia origin. Trends Neurosci..

[10] Surmeier D.J., Ding J., Day M., Wang Z., Shen W. (2007). D1 and D2 dopamine-receptor modulation of striatal glutamatergic signaling in striatal medium spiny neurons. Trends Neurosci..

[11] Liu C. (2020). Targeting the cholinergic system in Parkinson’s disease. Acta Pharm. Sin..

[12] Cho D.I., Zheng M., Kim K.M. (2010). Current perspectives on the selective regulation of dopamine D(2) and D(3) receptors. Arch. Pharm. Res..

[13] Seeman P. (1987). Dopamine receptors and the dopamine hypothesis of schizophrenia. Synapse.

[14] Kapur S., Zipursky R., Jones C., Remington G., Houle S. (2000). Relationship between dopamine D(2) occupancy, clinical response, and side effects: A double-blind PET study of first-episode schizophrenia. Am. J. Psychiatry.

[15] Ray R.D., Zald D.H. (2012). Anatomical insights into the interaction of emotion and cognition in the prefrontal cortex. Neurosci. Biobehav. Rev..

[16] Brisch R., Saniotis A., Wolf R., Bielau H., Bernstein H.G., Steiner J., Bogerts B., Braun K., Jankowski Z., Kumaratilake J. (2014). The role of dopamine in schizophrenia from a neurobiological and evolutionary perspective: Old fashioned, but still in vogue. Front. Psychiatry.

[17] Lee T., Seeman P., Rajput A., Farley I.J., Hornykiewicz O. (1978). Receptor basis for dopaminergic supersensitivity in Parkinson’s disease. Nature.

[18] Cunnah D., Besser M. (1991). Management of prolactinomas. Clin. Endocrinol..

[19] Kebabian J.W., Calne D.B. (1979). Multiple receptors for dopamine. Nature.

[20] De Camilli P., Macconi D., Spada A. (1979). Dopamine inhibits adenylate cyclase in human prolactin-secreting pituitary adenomas. Nature.

[21] Sunahara R.K., Niznik H.B., Weiner D.M., Stormann T.M., Brann M.R., Kennedy J.L., Gelernter J.E., Rozmahel R., Yang Y.L., Israel Y. (1990). Human dopamine D1 receptor encoded by an intronless gene on chromosome 5. Nature.

[22] Sunahara R.K., Guan H.C., O’Dowd B.F., Seeman P., Laurier L.G., Ng G., George S.R., Torchia J., Van Tol H.H., Niznik H.B. (1991). Cloning of the gene for a human dopamine D5 receptor with higher affinity for dopamine than D1. Nature.

[23] Bunzow J.R., Van Tol H.H., Grandy D.K., Albert P., Salon J., Christie M., Machida C.A., Neve K.A., Civelli O. (1988). Cloning and expression of a rat D2 dopamine receptor cDNA. Nature.

[24] Sokoloff P., Giros B., Martres M.P., Bouthenet M.L., Schwartz J.C. (1990). Molecular cloning and characterization of a novel dopamine receptor (D3) as a target for neuroleptics. Nature.

[25] Van Tol H.H., Bunzow J.R., Guan H.C., Sunahara R.K., Seeman P., Niznik H.B., Civelli O. (1991). Cloning of the gene for a human dopamine D4 receptor with high affinity for the antipsychotic clozapine. Nature.

[26] Franz D., Contreras F., Gonzalez H., Prado C., Elgueta D., Figueroa C., Pacheco R. (2015). Dopamine receptors D3 and D5 regulate CD4(+)T-cell activation and differentiation by modulating ERK activation and cAMP production. J. Neuroimmunol..

[27] Melnikov M., Rogovskii V., Boyko A., Pashenkov M. (2020). Dopaminergic Therapeutics in Multiple Sclerosis: Focus on Th17-Cell Functions. J. Neuroimmune Pharm..

[28] Hunger L., Kumar A., Schmidt R. (2020). Abundance Compensates Kinetics: Similar Effect of Dopamine Signals on D1 and D2 Receptor Populations. J. Neurosci. Off. J. Soc. Neurosci..

[29] Kim K.M., Nakajima S., Nakajima Y. (1997). Dopamine and GABA receptors in cultured substantia nigra neurons: Correlation of electrophysiology and immunocytochemistry. Neuroscience.

[30] Grace A.A., Bunney B.S. (1984). The control of firing pattern in nigral dopamine neurons: Burst firing. J. Neurosci. Off. J. Soc. Neurosci..

[31] Kim K.M., Nakajima Y., Nakajima S. (1995). G protein-coupled inward rectifier modulated by dopamine agonists in cultured substantia nigra neurons. Neuroscience.

[32] Yapo C., Nair A.G., Clement L., Castro L.R., Hellgren Kotaleski J., Vincent P. (2017). Detection of phasic dopamine by D1 and D2 striatal medium spiny neurons. J. Physiol..

[33] Marcott P.F., Gong S., Donthamsetti P., Grinnell S.G., Nelson M.N., Newman A.H., Birnbaumer L., Martemyanov K.A., Javitch J.A., Ford C.P. (2018). Regional Heterogeneity of D2-Receptor Signaling in the Dorsal Striatum and Nucleus Accumbens. Neuron.

[34] Arnsten A.F., Girgis R.R., Gray D.L., Mailman R.B. (2017). Novel Dopamine Therapeutics for Cognitive Deficits in Schizophrenia. Biol. Psychiatry.

[35] Hall H., Sedvall G., Magnusson O., Kopp J., Halldin C., Farde L. (1994). Distribution of D1- and D2-dopamine receptors, and dopamine and its metabolites in the human brain. Neuropsychopharmacol. Off. Publ. Am. Coll. Neuropsychopharmacol..

[36] Sawaguchi T., Goldman-Rakic P.S. (1991). D1 dopamine receptors in prefrontal cortex: Involvement in working memory. Science.

[37] Williams G.V., Goldman-Rakic P.S. (1995). Modulation of memory fields by dopamine D1 receptors in prefrontal cortex. Nature.

[38] Goldman-Rakic P.S. (1994). Working memory dysfunction in schizophrenia. J. Neuropsychiatry Clin. Neurosci..

[39] Kashima H. (1991). Frontal dysfunction of chronic schizophrenia—The pros and cons in neuropsychological assessment. Yakubutsu Seishin Kodo.

[40] Arnsten A.F., Wang M.J., Paspalas C.D. (2012). Neuromodulation of thought: Flexibilities and vulnerabilities in prefrontal cortical network synapses. Neuron.

[41] Mesulam M.M., Mufson E.J., Wainer B.H., Levey A.I. (1983). Central cholinergic pathways in the rat: An overview based on an alternative nomenclature (Ch1-Ch6). Neuroscience.

[42] Mesulam M.M., Mufson E.J., Levey A.I., Wainer B.H. (1983). Cholinergic innervation of cortex by the basal forebrain: Cytochemistry and cortical connections of the septal area, diagonal band nuclei, nucleus basalis (substantia innominata), and hypothalamus in the rhesus monkey. J. Comp. Neurol..

[43] Ballinger E.C., Ananth M., Talmage D.A., Role L.W. (2016). Basal Forebrain Cholinergic Circuits and Signaling in Cognition and Cognitive Decline. Neuron.

[44] Satoh K., Armstrong D.M., Fibiger H.C. (1983). A comparison of the distribution of central cholinergic neurons as demonstrated by acetylcholinesterase pharmacohistochemistry and choline acetyltransferase immunohistochemistry. Brain Res. Bull..

[45] Rye D.B., Saper C.B., Lee H.J., Wainer B.H. (1987). Pedunculopontine tegmental nucleus of the rat: Cytoarchitecture, cytochemistry, and some extrapyramidal connections of the mesopontine tegmentum. J. Comp. Neurol..

[46] Datta S., Siwek D.F. (1997). Excitation of the brain stem pedunculopontine tegmentum cholinergic cells induces wakefulness and REM sleep. J. Neurophysiol..

[47] Xiao C., Cho J.R., Zhou C., Treweek J.B., Chan K., McKinney S.L., Yang B., Gradinaru V. (2016). Cholinergic Mesopontine Signals Govern Locomotion and Reward through Dissociable Midbrain Pathways. Neuron.

[48] Ortells M.O., Lunt G.G. (1995). Evolutionary history of the ligand-gated ion-channel superfamily of receptors. Trends Neurosci..

[49] Corringer P.J., Le Novere N., Changeux J.P. (2000). Nicotinic receptors at the amino acid level. Annu. Rev. Pharm. Toxicol..

[50] Papke R.L., Brunzell D.H., De Biasi M. (2020). Cholinergic Receptors and Addiction. Curr. Top. Behav. Neurosci..

[51] Castro N.G., Albuquerque E.X. (1995). alpha-Bungarotoxin-sensitive hippocampal nicotinic receptor channel has a high calcium permeability. Biophys. J..

[52] Gray R., Rajan A.S., Radcliffe K.A., Yakehiro M., Dani J.A. (1996). Hippocampal synaptic transmission enhanced by low concentrations of nicotine. Nature.

[53] Liu L., Zhao-Shea R., McIntosh J.M., Gardner P.D., Tapper A.R. (2012). Nicotine persistently activates ventral tegmental area dopaminergic neurons via nicotinic acetylcholine receptors containing alpha4 and alpha6 subunits. Mol. Pharm..

[54] Grady S.R., Wageman C.R., Patzlaff N.E., Marks M.J. (2012). Low concentrations of nicotine differentially desensitize nicotinic acetylcholine receptors that include alpha5 or alpha6 subunits and that mediate synaptosomal neurotransmitter release. Neuropharmacology.

[55] Maskos U., Molles B.E., Pons S., Besson M., Guiard B.P., Guilloux J.P., Evrard A., Cazala P., Cormier A., Mameli-Engvall M. (2005). Nicotine reinforcement and cognition restored by targeted expression of nicotinic receptors. Nature.

[56] Pons S., Fattore L., Cossu G., Tolu S., Porcu E., McIntosh J.M., Changeux J.P., Maskos U., Fratta W. (2008). Crucial role of alpha4 and alpha6 nicotinic acetylcholine receptor subunits from ventral tegmental area in systemic nicotine self-administration. J. Neurosci. Off. J. Soc. Neurosci..

[57] Picciotto M.R., Zoli M. (2008). Neuroprotection via nAChRs: The role of nAChRs in neurodegenerative disorders such as Alzheimer’s and Parkinson’s disease. Front. Biosci..

[58] Brunzell D.H., McIntosh J.M., Papke R.L. (2014). Diverse strategies targeting alpha7 homomeric and alpha6beta2* heteromeric nicotinic acetylcholine receptors for smoking cessation. Ann. N. Y. Acad. Sci..

[59] Melis M., Scheggi S., Carta G., Madeddu C., Lecca S., Luchicchi A., Cadeddu F., Frau R., Fattore L., Fadda P. (2013). PPARalpha regulates cholinergic-driven activity of midbrain dopamine neurons via a novel mechanism involving alpha7 nicotinic acetylcholine receptors. J. Neurosci. Off. J. Soc. Neurosci..

[60] Jackson A., Bagdas D., Muldoon P.P., Lichtman A.H., Carroll F.I., Greenwald M., Miles M.F., Damaj M.I. (2017). In vivo interactions between alpha7 nicotinic acetylcholine receptor and nuclear peroxisome proliferator-activated receptor-alpha: Implication for nicotine dependence. Neuropharmacology.

[61] Corrigall W.A., Coen K.M., Adamson K.L. (1994). Self-administered nicotine activates the mesolimbic dopamine system through the ventral tegmental area. Brain Res..

[62] Gotti C., Zoli M., Clementi F. (2006). Brain nicotinic acetylcholine receptors: Native subtypes and their relevance. Trends Pharm. Sci..

[63] Mineur Y.S., Brunzell D.H., Grady S.R., Lindstrom J.M., McIntosh J.M., Marks M.J., King S.L., Picciotto M.R. (2009). Localized low-level re-expression of high-affinity mesolimbic nicotinic acetylcholine receptors restores nicotine-induced locomotion but not place conditioning. Genes Brain Behav..

[64] Charpantier E., Barneoud P., Moser P., Besnard F., Sgard F. (1998). Nicotinic acetylcholine subunit mRNA expression in dopaminergic neurons of the rat substantia nigra and ventral tegmental area. Neuroreport.

[65] Cui C., Booker T.K., Allen R.S., Grady S.R., Whiteaker P., Marks M.J., Salminen O., Tritto T., Butt C.M., Allen W.R. (2003). The beta3 nicotinic receptor subunit: A component of alpha-conotoxin MII-binding nicotinic acetylcholine receptors that modulate dopamine release and related behaviors. J. Neurosci. Off. J. Soc. Neurosci..

[66] Grady S.R., Salminen O., Laverty D.C., Whiteaker P., McIntosh J.M., Collins A.C., Marks M.J. (2007). The subtypes of nicotinic acetylcholine receptors on dopaminergic terminals of mouse striatum. Biochem. Pharm..

[67] Exley R., Cragg S.J. (2008). Presynaptic nicotinic receptors: A dynamic and diverse cholinergic filter of striatal dopamine neurotransmission. Br. J. Pharm..

[68] Fratiglioni L., Wang H.X. (2000). Smoking and Parkinson’s and Alzheimer’s disease: Review of the epidemiological studies. Behav. Brain Res..

[69] Quik M., McIntosh J.M. (2006). Striatal alpha6* nicotinic acetylcholine receptors: Potential targets for Parkinson’s disease therapy. J. Pharmacol. Exp. Ther..

[70] Collo G., Bono F., Cavalleri L., Plebani L., Mitola S., Merlo Pich E., Millan M.J., Zoli M., Maskos U., Spano P. (2013). Nicotine-induced structural plasticity in mesencephalic dopaminergic neurons is mediated by dopamine D3 receptors and Akt-mTORC1 signaling. Mol. Pharm..

[71] Zoli M., Pistillo F., Gotti C. (2015). Diversity of native nicotinic receptor subtypes in mammalian brain. Neuropharmacology.

[72] Champtiaux N., Gotti C., Cordero-Erausquin M., David D.J., Przybylski C., Lena C., Clementi F., Moretti M., Rossi F.M., Le Novere N. (2003). Subunit composition of functional nicotinic receptors in dopaminergic neurons investigated with knock-out mice. J. Neurosci. Off. J. Soc. Neurosci..

[73] Le Foll B., Diaz J., Sokoloff P. (2003). Increased dopamine D3 receptor expression accompanying behavioral sensitization to nicotine in rats. Synapse.

[74] Patel K.R., Cherian J., Gohil K., Atkinson D. (2014). Schizophrenia: Overview and treatment options. Pharm. Ther..

[75] Picchioni M.M., Murray R.M. (2007). Schizophrenia. BMJ.

[76] Creese I., Burt D.R., Snyder S.H. (1976). Dopamine receptor binding predicts clinical and pharmacological potencies of antischizophrenic drugs. Science.

[77] Meltzer H.Y., Stahl S.M. (1976). The dopamine hypothesis of schizophrenia: A review. Schizophr. Bull..

[78] Abi-Dargham A. (2004). Do we still believe in the dopamine hypothesis? New data bring new evidence. Int. J. Neuropsychopharmacol..

[79] Sokoloff P., Le Foll B. (2017). The dopamine D3 receptor, a quarter century later. Eur. J. Neurosci..

[80] Alasmari F., Goodwani S., McCullumsmith R.E., Sari Y. (2018). Role of glutamatergic system and mesocorticolimbic circuits in alcohol dependence. Prog. Neurobiol..

[81] Schwartz T.L., Sachdeva S., Stahl S.M. (2012). Glutamate neurocircuitry: Theoretical underpinnings in schizophrenia. Front. Pharm..

[82] Zhang D., Gao M., Xu D., Shi W.X., Gutkin B.S., Steffensen S.C., Lukas R.J., Wu J. (2012). Impact of prefrontal cortex in nicotine-induced excitation of ventral tegmental area dopamine neurons in anesthetized rats. J. Neurosci. Off. J. Soc. Neurosci..

[83] Goff D.C., Henderson D.C., Amico E. (1992). Cigarette smoking in schizophrenia: Relationship to psychopathology and medication side effects. Am. J. Psychiatry.

[84] Ziedonis D.M., Kosten T.R., Glazer W.M., Frances R.J. (1994). Nicotine dependence and schizophrenia. Hosp. Community Psychiatry.

[85] Freedman R., Adams C.E., Leonard S. (2000). The alpha7-nicotinic acetylcholine receptor and the pathology of hippocampal interneurons in schizophrenia. J. Chem. Neuroanat..

[86] Durany N., Zochling R., Boissl K.W., Paulus W., Ransmayr G., Tatschner T., Danielczyk W., Jellinger K., Deckert J., Riederer P. (2000). Human post-mortem striatal alpha4beta2 nicotinic acetylcholine receptor density in schizophrenia and Parkinson’s syndrome. Neurosci. Lett..

[87] Patkar A.A., Gopalakrishnan R., Lundy A., Leone F.T., Certa K.M., Weinstein S.P. (2002). Relationship between tobacco smoking and positive and negative symptoms in schizophrenia. J. Nerv. Ment. Dis..

[88] Krishnadas R., Jauhar S., Telfer S., Shivashankar S., McCreadie R.G. (2012). Nicotine dependence and illness severity in schizophrenia. Br. J. Psychiatry.

[89] Basu A., Nebhinani N. (2013). Nicotine dependence in patients with schizophrenia. Br. J. Psychiatry.

[90] Coyle J.T. (1996). The glutamatergic dysfunction hypothesis for schizophrenia. Harv. Rev. Psychiatry.

[91] Halberstadt A.L. (1995). The phencyclidine-glutamate model of schizophrenia. Clin. Neuropharmacol..

[92] Krystal J.H., Karper L.P., Seibyl J.P., Freeman G.K., Delaney R., Bremner J.D., Heninger G.R., Bowers M.B., Charney D.S. (1994). Subanesthetic effects of the noncompetitive NMDA antagonist, ketamine, in humans. Psychotomimetic, perceptual, cognitive, and neuroendocrine responses. Arch. Gen. Psychiatry.

[93] Hu W., MacDonald M.L., Elswick D.E., Sweet R.A. (2015). The glutamate hypothesis of schizophrenia: Evidence from human brain tissue studies. Ann. N. Y. Acad. Sci..

[94] Paz R.D., Tardito S., Atzori M., Tseng K.Y. (2008). Glutamatergic dysfunction in schizophrenia: From basic neuroscience to clinical psychopharmacology. Eur. Neuropsychopharmacol..

[95] Kristiansen L.V., Huerta I., Beneyto M., Meador-Woodruff J.H. (2007). NMDA receptors and schizophrenia. Curr. Opin. Pharm..

[96] Catts V.S., Lai Y.L., Weickert C.S., Weickert T.W., Catts S.V. (2016). A quantitative review of the postmortem evidence for decreased cortical N-methyl-D-aspartate receptor expression levels in schizophrenia: How can we link molecular abnormalities to mismatch negativity deficits?. Biol. Psychol..

[97] Datta D., Arnsten A.F.T. (2018). Unique Molecular Regulation of Higher-Order Prefrontal Cortical Circuits: Insights into the Neurobiology of Schizophrenia. ACS Chem. Neurosci..

[98] Stahl S.M. (2007). Beyond the dopamine hypothesis to the NMDA glutamate receptor hypofunction hypothesis of schizophrenia. CNS Spectr..

[99] Davis K.L., Kahn R.S., Ko G., Davidson M. (1991). Dopamine in schizophrenia: A review and reconceptualization. Am. J. Psychiatry.

[100] Weinberger D.R. (1987). Implications of normal brain development for the pathogenesis of schizophrenia. Arch. Gen. Psychiatry.

[101] Friston K.J., Frith C.D. (1995). Schizophrenia: A disconnection syndrome?. Clin. Neurosci..

[102] Antonova E., Sharma T., Morris R., Kumari V. (2004). The relationship between brain structure and neurocognition in schizophrenia: A selective review. Schizophr. Res..

[103] Garey L.J., Ong W.Y., Patel T.S., Kanani M., Davis A., Mortimer A.M., Barnes T.R., Hirsch S.R. (1998). Reduced dendritic spine density on cerebral cortical pyramidal neurons in schizophrenia. J. Neurol. Neurosurg. Psychiatry.

[104] Wahl-Schott C., Biel M. (2009). HCN channels: Structure, cellular regulation and physiological function. Cell. Mol. Life Sci. CMLS.

[105] Garden D.L., Dodson P.D., O’Donnell C., White M.D., Nolan M.F. (2008). Tuning of synaptic integration in the medial entorhinal cortex to the organization of grid cell firing fields. Neuron.

[106] Kase D., Imoto K. (2012). The Role of HCN Channels on Membrane Excitability in the Nervous System. J. Signal. Transduct..

[107] Arnsten A.F., Wang M., Paspalas C.D. (2015). Dopamine’s Actions in Primate Prefrontal Cortex: Challenges for Treating Cognitive Disorders. Pharmacol. Rev..

[108] Silva-Gomez A.B., Rojas D., Juarez I., Flores G. (2003). Decreased dendritic spine density on prefrontal cortical and hippocampal pyramidal neurons in postweaning social isolation rats. Brain Res..

[109] Tregellas J.R., Wylie K.P. (2019). Alpha7 Nicotinic Receptors as Therapeutic Targets in Schizophrenia. Nicotine Tob. Res..

[110] Gee K.W., Olincy A., Kanner R., Johnson L., Hogenkamp D., Harris J., Tran M., Edmonds S.A., Sauer W., Yoshimura R. (2017). First in human trial of a type I positive allosteric modulator of alpha7-nicotinic acetylcholine receptors: Pharmacokinetics, safety, and evidence for neurocognitive effect of AVL-3288. J. Psychopharmacol..

[111] Kem W.R., Olincy A., Johnson L., Harris J., Wagner B.D., Buchanan R.W., Christians U., Freedman R. (2018). Pharmacokinetic Limitations on Effects of an Alpha7-Nicotinic Receptor Agonist in Schizophrenia: Randomized Trial with an Extended-Release Formulation. Neuropsychopharmacol. Off. Publ. Am. Coll. Neuropsychopharmacol..

[112] Kitagawa H., Takenouchi T., Azuma R., Wesnes K.A., Kramer W.G., Clody D.E., Burnett A.L. (2003). Safety, pharmacokinetics, and effects on cognitive function of multiple doses of GTS-21 in healthy, male volunteers. Neuropsychopharmacol. Off. Publ. Am. Coll. Neuropsychopharmacol..

[113] Vijverman A.C., Fox S.H. (2014). New treatments for the motor symptoms of Parkinson’s disease. Expert Rev. Clin. Pharm..

[114] LeWitt P.A., Fahn S. (2016). Levodopa therapy for Parkinson disease: A look backward and forward. Neurology.

[115] Zhang X., Gao F., Wang D., Li C., Fu Y., He W., Zhang J. (2018). Tau Pathology in Parkinson’s Disease. Front. Neurol..

[116] Smith W.W., Pei Z., Jiang H., Dawson V.L., Dawson T.M., Ross C.A. (2006). Kinase activity of mutant LRRK2 mediates neuronal toxicity. Nat. Neurosci..

[117] West A.B., Moore D.J., Choi C., Andrabi S.A., Li X., Dikeman D., Biskup S., Zhang Z., Lim K.L., Dawson V.L. (2007). Parkinson’s disease-associated mutations in LRRK2 link enhanced GTP-binding and kinase activities to neuronal toxicity. Hum. Mol. Genet..

[118] Kitada T., Asakawa S., Hattori N., Matsumine H., Yamamura Y., Minoshima S., Yokochi M., Mizuno Y., Shimizu N. (1998). Mutations in the parkin gene cause autosomal recessive juvenile parkinsonism. Nature.

[119] Itier J.M., Ibanez P., Mena M.A., Abbas N., Cohen-Salmon C., Bohme G.A., Laville M., Pratt J., Corti O., Pradier L. (2003). Parkin gene inactivation alters behaviour and dopamine neurotransmission in the mouse. Hum. Mol. Genet..

[120] Palacino J.J., Sagi D., Goldberg M.S., Krauss S., Motz C., Wacker M., Klose J., Shen J. (2004). Mitochondrial dysfunction and oxidative damage in parkin-deficient mice. J. Biol. Chem..

[121] Kim R.H., Smith P.D., Aleyasin H., Hayley S., Mount M.P., Pownall S., Wakeham A., You-Ten A.J., Kalia S.K., Horne P. (2005). Hypersensitivity of DJ-1-deficient mice to 1-methyl-4-phenyl-1,2,3,6-tetrahydropyrindine (MPTP) and oxidative stress. Proc. Natl. Acad. Sci. USA.

[122] Meulener M.C., Xu K., Thomson L., Ischiropoulos H., Bonini N.M. (2006). Mutational analysis of DJ-1 in Drosophila implicates functional inactivation by oxidative damage and aging. Proc. Natl. Acad. Sci. USA.

[123] Clark I.E., Dodson M.W., Jiang C., Cao J.H., Huh J.R., Seol J.H., Yoo S.J., Hay B.A., Guo M. (2006). Drosophila pink1 is required for mitochondrial function and interacts genetically with parkin. Nature.

[124] Yang Y., Gehrke S., Imai Y., Huang Z., Ouyang Y., Wang J.W., Yang L., Beal M.F., Vogel H., Lu B. (2006). Mitochondrial pathology and muscle and dopaminergic neuron degeneration caused by inactivation of Drosophila Pink1 is rescued by Parkin. Proc. Natl. Acad. Sci. USA.

[125] Xu M., Moratalla R., Gold L.H., Hiroi N., Koob G.F., Graybiel A.M., Tonegawa S. (1994). Dopamine D1 receptor mutant mice are deficient in striatal expression of dynorphin and in dopamine-mediated behavioral responses. Cell.

[126] Gantois I., Fang K., Jiang L., Babovic D., Lawrence A.J., Ferreri V., Teper Y., Jupp B., Ziebell J., Morganti-Kossmann C.M. (2007). Ablation of D1 dopamine receptor-expressing cells generates mice with seizures, dystonia, hyperactivity, and impaired oral behavior. Proc. Natl. Acad. Sci. USA.

[127] Usiello A., Baik J.H., Rouge-Pont F., Picetti R., Dierich A., LeMeur M., Piazza P.V., Borrelli E. (2000). Distinct functions of the two isoforms of dopamine D2 receptors. Nature.

[128] Xu R., Hranilovic D., Fetsko L.A., Bucan M., Wang Y. (2002). Dopamine D2S and D2L receptors may differentially contribute to the actions of antipsychotic and psychotic agents in mice. Mol. Psychiatry.

[129] Tinsley R.B., Bye C.R., Parish C.L., Tziotis-Vais A., George S., Culvenor J.G., Li Q.X., Masters C.L., Finkelstein D.I., Horne M.K. (2009). Dopamine D2 receptor knockout mice develop features of Parkinson disease. Ann. Neurol..

[130] Nagai Y., Ueno S., Saeki Y., Soga F., Hirano M., Yanagihara T. (1996). Decrease of the D3 dopamine receptor mRNA expression in lymphocytes from patients with Parkinson’s disease. Neurology.

[131] Caronti B., Antonini G., Calderaro C., Ruggieri S., Palladini G., Pontieri F.E., Colosimo C. (2001). Dopamine transporter immunoreactivity in peripheral blood lymphocytes in Parkinson’s disease. J. Neural. Transm..

[132] Yang P., Perlmutter J.S., Benzinger T.L.S., Morris J.C., Xu J. (2020). Dopamine D3 receptor: A neglected participant in Parkinson Disease pathogenesis and treatment?. Ageing Res. Rev..

[133] Favier M., Carcenac C., Drui G., Vachez Y., Boulet S., Savasta M., Carnicella S. (2017). Implication of dorsostriatal D3 receptors in motivational processes: A potential target for neuropsychiatric symptoms in Parkinson’s disease. Sci. Rep..

[134] Rubinstein M., Phillips T.J., Bunzow J.R., Falzone T.L., Dziewczapolski G., Zhang G., Fang Y., Larson J.L., McDougall J.A., Chester J.A. (1997). Mice lacking dopamine D4 receptors are supersensitive to ethanol, cocaine, and methamphetamine. Cell.

[135] Dulawa S.C., Grandy D.K., Low M.J., Paulus M.P., Geyer M.A. (1999). Dopamine D4 receptor-knock-out mice exhibit reduced exploration of novel stimuli. J. Neurosci. Off. J. Soc. Neurosci..

[136] Falzone T.L., Gelman D.M., Young J.I., Grandy D.K., Low M.J., Rubinstein M. (2002). Absence of dopamine D4 receptors results in enhanced reactivity to unconditioned, but not conditioned, fear. Eur. J. Neurosci..

[137] Cormier F., Muellner J., Corvol J.C. (2013). Genetics of impulse control disorders in Parkinson’s disease. J. Neural. Transm..

[138] Tanimura A., Pancani T., Lim S.A.O., Tubert C., Melendez A.E., Shen W., Surmeier D.J. (2018). Striatal cholinergic interneurons and Parkinson’s disease. Eur. J. Neurosci..

[139] Zhou F.M., Wilson C.J., Dani J.A. (2002). Cholinergic interneuron characteristics and nicotinic properties in the striatum. J. Neurobiol..

[140] Woolf N.J., Butcher L.L. (1981). Cholinergic neurons in the caudate-putamen complex proper are intrinsically organized: A combined Evans blue and acetylcholinesterase analysis. Brain Res. Bull..

[141] Bolam J.P. (1984). Synapses of identified neurons in the neostriatum. Ciba Found. Symp..

[142] Contant C., Umbriaco D., Garcia S., Watkins K.C., Descarries L. (1996). Ultrastructural characterization of the acetylcholine innervation in adult rat neostriatum. Neuroscience.

[143] Fonnum F. (1973). Recent developments in biochemical investigations of cholinergic transmission. Brain Res..

[144] Ikarashi Y., Yuzurihara M., Takahashi A., Hirohisa I., Shiobara T., Maruyama Y. (1999). Modulation of acetylcholine release via GABAA and GABAB receptors in rat striatum. Brain Res..

[145] Marti M., Paganini F., Stocchi S., Bianchi C., Beani L., Morari M. (2001). Presynaptic group I and II metabotropic glutamate receptors oppositely modulate striatal acetylcholine release. Eur. J. Neurosci..

[146] Lim S.A., Kang U.J., McGehee D.S. (2014). Striatal cholinergic interneuron regulation and circuit effects. Front. Synaptic Neurosci..

[147] Acquas E., Di Chiara G. (2001). Role of dopamine D1 receptors in the control of striatal acetylcholine release by endogenous dopamine. Neurol. Sci..

[148] Damsma G., Tham C.S., Robertson G.S., Fibiger H.C. (1990). Dopamine D1 receptor stimulation increases striatal acetylcholine release in the rat. Eur. J. Pharm..

[149] Faure P., Tolu S., Valverde S., Naude J. (2014). Role of nicotinic acetylcholine receptors in regulating dopamine neuron activity. Neuroscience.

[150] Zhou F.M., Liang Y., Dani J.A. (2001). Endogenous nicotinic cholinergic activity regulates dopamine release in the striatum. Nat. Neurosci..

[151] Quik M., Wonnacott S. (2011). alpha6beta2* and alpha4beta2* nicotinic acetylcholine receptors as drug targets for Parkinson’s disease. Pharmacol. Rev..

[152] Bordia T., Grady S.R., McIntosh J.M., Quik M. (2007). Nigrostriatal damage preferentially decreases a subpopulation of alpha6beta2* nAChRs in mouse, monkey, and Parkinson’s disease striatum. Mol. Pharm..

[153] Perez X.A., Bordia T., McIntosh J.M., Grady S.R., Quik M. (2008). Long-term nicotine treatment differentially regulates striatal alpha6alpha4beta2* and alpha6(nonalpha4)beta2* nAChR expression and function. Mol. Pharm..

[154] Kaiser S., Wonnacott S. (2000). alpha-bungarotoxin-sensitive nicotinic receptors indirectly modulate [(3)H]dopamine release in rat striatal slices via glutamate release. Mol. Pharm..

[155] Quik M., Cox H., Parameswaran N., O’Leary K., Langston J.W., Di Monte D. (2007). Nicotine reduces levodopa-induced dyskinesias in lesioned monkeys. Ann. Neurol..

[156] O’Neill M.J., Murray T.K., Lakics V., Visanji N.P., Duty S. (2002). The role of neuronal nicotinic acetylcholine receptors in acute and chronic neurodegeneration. Curr. Drug Targets CNS Neurol. Disord..

[157] Elbaz A., Moisan F. (2008). Update in the epidemiology of Parkinson’s disease. Curr. Opin. Neurol..

[158] Allam M.F., Campbell M.J., Hofman A., Del Castillo A.S., Fernandez-Crehuet Navajas R. (2004). Smoking and Parkinson’s disease: Systematic review of prospective studies. Mov. Disord..

[159] Quik M., Boyd J.T., Bordia T., Perez X. (2019). Potential Therapeutic Application for Nicotinic Receptor Drugs in Movement Disorders. Nicotine Tob. Res..

[160] Tanner C.M., Goldman S.M., Aston D.A., Ottman R., Ellenberg J., Mayeux R., Langston J.W. (2002). Smoking and Parkinson’s disease in twins. Neurology.

[161] Quik M., Huang L.Z., Parameswaran N., Bordia T., Campos C., Perez X.A. (2009). Multiple roles for nicotine in Parkinson’s disease. Biochem. Pharm..

[162] Zoli M., Moretti M., Zanardi A., McIntosh J.M., Clementi F., Gotti C. (2002). Identification of the nicotinic receptor subtypes expressed on dopaminergic terminals in the rat striatum. J. Neurosci. Off. J. Soc. Neurosci..

[163] Pahwa R., Tanner C.M., Hauser R.A., Isaacson S.H., Nausieda P.A., Truong D.D., Agarwal P., Hull K.L., Lyons K.E., Johnson R. (2017). ADS-5102 (Amantadine) Extended-Release Capsules for Levodopa-Induced Dyskinesia in Parkinson Disease (EASE LID Study): A Randomized Clinical Trial. JAMA Neurol..

[164] Du H., Nie S., Chen G., Ma K., Xu Y., Zhang Z., Papa S.M., Cao X. (2015). Levetiracetam Ameliorates L-DOPA-Induced Dyskinesia in Hemiparkinsonian Rats Inducing Critical Molecular Changes in the Striatum. Parkinsons Dis..

[165] Bordia T., Campos C., Huang L., Quik M. (2008). Continuous and intermittent nicotine treatment reduces L-3,4-dihydroxyphenylalanine (L-DOPA)-induced dyskinesias in a rat model of Parkinson’s disease. J. Pharmacol. Exp. Ther..

[166] Feyder M., Bonito-Oliva A., Fisone G. (2011). L-DOPA-Induced Dyskinesia and Abnormal Signaling in Striatal Medium Spiny Neurons: Focus on Dopamine D1 Receptor-Mediated Transmission. Front. Behav. Neurosci..

[167] Aubert I., Guigoni C., Hakansson K., Li Q., Dovero S., Barthe N., Bioulac B.H., Gross C.E., Fisone G., Bloch B. (2005). Increased D1 dopamine receptor signaling in levodopa-induced dyskinesia. Ann. Neurol..

[168] Bastide M.F., Meissner W.G., Picconi B., Fasano S., Fernagut P.O., Feyder M., Francardo V., Alcacer C., Ding Y., Brambilla R. (2015). Pathophysiology of L-dopa-induced motor and non-motor complications in Parkinson’s disease. Prog. Neurobiol..

[169] Huot P., Johnston T.H., Koprich J.B., Fox S.H., Brotchie J.M. (2013). The pharmacology of L-DOPA-induced dyskinesia in Parkinson’s disease. Pharmacol. Rev..

[170] Huang L.Z., Grady S.R., Quik M. (2011). Nicotine reduces L-DOPA-induced dyskinesias by acting at beta2* nicotinic receptors. J. Pharmacol. Exp. Ther..

[171] Quik M., Mallela A., Chin M., McIntosh J.M., Perez X.A., Bordia T. (2013). Nicotine-mediated improvement in L-dopa-induced dyskinesias in MPTP-lesioned monkeys is dependent on dopamine nerve terminal function. Neurobiol. Dis..

[172] Zhang D., McGregor M., Bordia T., Perez X.A., McIntosh J.M., Decker M.W., Quik M. (2015). alpha7 nicotinic receptor agonists reduce levodopa-induced dyskinesias with severe nigrostriatal damage. Mov. Disord..

[173] Picciotto M.R., Addy N.A., Mineur Y.S., Brunzell D.H. (2008). It is not “either/or”: Activation and desensitization of nicotinic acetylcholine receptors both contribute to behaviors related to nicotine addiction and mood. Prog. Neurobiol..

[174] Buccafusco J.J., Beach J.W., Terry A.V. (2009). Desensitization of nicotinic acetylcholine receptors as a strategy for drug development. J. Pharmacol. Exp. Ther..

[175] Won L., Ding Y., Singh P., Kang U.J. (2014). Striatal cholinergic cell ablation attenuates L-DOPA induced dyskinesia in Parkinsonian mice. J. Neurosci. Off. J. Soc. Neurosci..

[176] Bordia T., Perez X.A., Heiss J., Zhang D., Quik M. (2016). Optogenetic activation of striatal cholinergic interneurons regulates L-dopa-induced dyskinesias. Neurobiol. Dis..

[177] Kihara T., Shimohama S., Sawada H., Honda K., Nakamizo T., Shibasaki H., Kume T., Akaike A. (2001). alpha 7 nicotinic receptor transduces signals to phosphatidylinositol 3-kinase to block A beta-amyloid-induced neurotoxicity. J. Biol. Chem..

[178] Shimohama S., Kihara T. (2001). Nicotinic receptor-mediated protection against beta-amyloid neurotoxicity. Biol. Psychiatry.

[179] Shaw S., Bencherif M., Marrero M.B. (2002). Janus kinase 2, an early target of alpha 7 nicotinic acetylcholine receptor-mediated neuroprotection against Abeta-(1-42) amyloid. J. Biol. Chem..

[180] Toborek M., Son K.W., Pudelko A., King-Pospisil K., Wylegala E., Malecki A. (2007). ERK 1/2 signaling pathway is involved in nicotine-mediated neuroprotection in spinal cord neurons. J. Cell. Biochem..

[181] Ren K., Puig V., Papke R.L., Itoh Y., Hughes J.A., Meyer E.M. (2005). Multiple calcium channels and kinases mediate alpha7 nicotinic receptor neuroprotection in PC12 cells. J. Neurochem..

[182] Ren K., King M.A., Liu J., Siemann J., Altman M., Meyers C., Hughes J.A., Meyer E.M. (2007). The alpha7 nicotinic receptor agonist 4OH-GTS-21 protects axotomized septohippocampal cholinergic neurons in wild type but not amyloid-overexpressing transgenic mice. Neuroscience.

[183] Tyagi E., Agrawal R., Nath C., Shukla R. (2010). Cholinergic protection via alpha7 nicotinic acetylcholine receptors and PI3K-Akt pathway in LPS-induced neuroinflammation. Neurochem. Int..

[184] Parada E., Egea J., Romero A., del Barrio L., Garcia A.G., Lopez M.G. (2010). Poststress treatment with PNU282987 can rescue SH-SY5Y cells undergoing apoptosis via alpha7 nicotinic receptors linked to a Jak2/Akt/HO-1 signaling pathway. Free Radic. Biol. Med..

[185] Lawrence T., Natoli G. (2011). Transcriptional regulation of macrophage polarization: Enabling diversity with identity. Nat. Rev. Immunol..

[186] Roszer T. (2015). Understanding the Mysterious M2 Macrophage through Activation Markers and Effector Mechanisms. Mediat. Inflamm..

[187] Qin H., Buckley J.A., Li X., Liu Y., Fox T.H., Meares G.P., Yu H., Yan Z., Harms A.S., Li Y. (2016). Inhibition of the JAK/STAT Pathway Protects Against alpha-Synuclein-Induced Neuroinflammation and Dopaminergic Neurodegeneration. J. Neurosci. Off. J. Soc. Neurosci..

[188] Yan A., Zhang Y., Lin J., Song L., Wang X., Liu Z. (2018). Partial Depletion of Peripheral M1 Macrophages Reverses Motor Deficits in MPTP-Treated Mouse by Suppressing Neuroinflammation and Dopaminergic Neurodegeneration. Front. Aging Neurosci..

[189] Yang D.C., Chen C.H. (2018). Cigarette Smoking-Mediated Macrophage Reprogramming: Mechanistic Insights and Therapeutic Implications. J. Nat. Sci..

[190] Zhang Q., Lu Y., Bian H., Guo L., Zhu H. (2017). Activation of the alpha7 nicotinic receptor promotes lipopolysaccharide-induced conversion of M1 microglia to M2. Am. J. Transl. Res..

[191] Hu X., Li P., Guo Y., Wang H., Leak R.K., Chen S., Gao Y., Chen J. (2012). Microglia/macrophage polarization dynamics reveal novel mechanism of injury expansion after focal cerebral ischemia. Stroke.

[192] Chen W., Wang J., Jia L., Liu J., Tian Y. (2016). Attenuation of the programmed cell death-1 pathway increases the M1 polarization of macrophages induced by zymosan. Cell Death Dis..

[193] Kanazawa M., Ninomiya I., Hatakeyama M., Takahashi T., Shimohata T. (2017). Microglia and Monocytes/Macrophages Polarization Reveal Novel Therapeutic Mechanism against Stroke. Int. J. Mol. Sci..

[194] Kawabori M., Kacimi R., Kauppinen T., Calosing C., Kim J.Y., Hsieh C.L., Nakamura M.C., Yenari M.A. (2015). Triggering receptor expressed on myeloid cells 2 (TREM2) deficiency attenuates phagocytic activities of microglia and exacerbates ischemic damage in experimental stroke. J. Neurosci. Off. J. Soc. Neurosci..

[195] Belluardo N., Mudo G., Blum M., Fuxe K. (2000). Central nicotinic receptors, neurotrophic factors and neuroprotection. Behav. Brain Res..

[196] Ward R.J., Lallemand F., de Witte P., Dexter D.T. (2008). Neurochemical pathways involved in the protective effects of nicotine and ethanol in preventing the development of Parkinson’s disease: Potential targets for the development of new therapeutic agents. Prog. Neurobiol..

[197] Shimohama S. (2009). Nicotinic receptor-mediated neuroprotection in neurodegenerative disease models. Biol. Pharm. Bull..

[198] Quik M., Perez X.A., Bordia T. (2012). Nicotine as a potential neuroprotective agent for Parkinson’s disease. Mov. Disord..

[199] Castagnoli K., Petzer J.B., Steyn S.J., van der Schyf C.J., Castagnoli N. (2003). Inhibition of human MAO-A and MAO-B by a compound isolated from flue-cured tobacco leaves and its neuroprotective properties in the MPTP mouse model of neurodegeneration. Inflammopharmacology.

[200] Lezi E., Swerdlow R.H. (2012). Mitochondria in neurodegeneration. Adv. Exp. Med. Biol..

[201] Reddy P.H. (2009). Role of mitochondria in neurodegenerative diseases: Mitochondria as a therapeutic target in Alzheimer’s disease. CNS Spectr..

[202] Malinska D., Wieckowski M.R., Michalska B., Drabik K., Prill M., Patalas-Krawczyk P., Walczak J., Szymanski J., Mathis C., Van der Toorn M. (2019). Mitochondria as a possible target for nicotine action. J. Bioenerg. Biomembr..

[203] Benowitz N.L., Hukkanen J., Jacob P. (2009). Nicotine chemistry, metabolism, kinetics and biomarkers. Handb. Exp. Pharm..

[204] Cormier A., Morin C., Zini R., Tillement J.P., Lagrue G. (2003). Nicotine protects rat brain mitochondria against experimental injuries. Neuropharmacology.

[205] Newman M.B., Arendash G.W., Shytle R.D., Bickford P.C., Tighe T., Sanberg P.R. (2002). Nicotine’s oxidative and antioxidant properties in CNS. Life Sci..

[206] Xie Y.X., Bezard E., Zhao B.L. (2005). Investigating the receptor-independent neuroprotective mechanisms of nicotine in mitochondria. J. Biol. Chem..

[207] Acharya S., Kundu D., Choi H.J., Kim K.M. (2020). Metabotropic signaling cascade involved in alpha4beta2 nicotinic acetylcholine receptor-mediated PKCbetaII activation. Biochim. Biophys. Acta Mol. Cell Res..

